# Draft Genome Sequence of an Enterococcus faecalis Strain (24FS) That Was Isolated from Healthy Infant Feces and Exhibits High Antibacterial Activity, Multiple-Antibiotic Resistance, and Multiple Virulence Factors

**DOI:** 10.1128/MRA.00047-19

**Published:** 2019-03-28

**Authors:** Nancy M. El Halfawy, Moustafa Y. El-Naggar, Simon C. Andrews

**Affiliations:** a Botany and Microbiology Department, Faculty of Science, Alexandria University, Alexandria, Egypt; b School of Biological Sciences, University of Reading, Reading, United Kingdom; University of California, Riverside

## Abstract

Enterococcus faecalis 24FS is a bacteriocin-producing, multiply antibiotic-resistant, and potentially virulent bacterium isolated from healthy infant feces. The draft 2.9-Mb genome sequence revealed 2,968 protein-encoding genes; 11 antibiotic resistance, 8 virulence, and 3 bacteriocin genes; and 2 plasmids, 4 prophages, 30 insertion sequence (IS) elements, 1 transposon, and 1 integron.

## ANNOUNCEMENT

Enterococcus faecalis is a common commensal of the human intestine (1, 2) but is also a frequent cause of hospital-acquired enterococcal infections (3). An E. faecalis fecal isolate (24FS) from a healthy 6-month-old infant (Alexandria, Egypt) displayed multiple-antibiotic resistance, strong antibacterial properties, and numerous virulence factors and thus provided the opportunity to study the emergence of pathogenic/resistance properties in a nonclinical strain from a developing nation.

Strain 24FS was isolated from infant feces (in 2016) on bile esculin azide agar. Genomic DNA was isolated using a GeneJET purification kit. Genome sequencing was performed by MicrobesNG (Birmingham, UK) on MiSeq and HiSeq 2500 platforms (Illumina) with 30× sequence coverage, giving 924,992 reads with a median insert size of 529 bases and a 136-fold coverage of the genome. The reads were trimmed using Trimmomatic (4) (v 0.38) by identification of adapter sequences, and the quality of trimmed reads was assessed using in-house scripts combined with the BWA-MEM software (5) (v 0.7.9). *De novo* assembly was performed using SPAdes (6) (v 3.7.0), yielding 46 contigs of more than 1,000 bp. Assembly quality was assessed using QUAST (7). The 2,984,798-bp draft genome has 2,968 protein-encoding genes, 54 tRNA genes, and 111 pseudogenes and a G+C content of 37.38%, as predicted with Rapid Annotations using Subsystem Technology (RAST) (8, 9).

E. faecalis 24FS showed a broad-spectrum antibacterial activity (in spot on the lawn assays [10]), inhibiting the pathogens Listeria monocytogenes 10403S, Salmonella enterica PT4, Staphylococcus aureus, and Pseudomonas aeruginosa PAO1. The PATtyFam algorithm identified three bacteriocin genes encoding a colicin V (20,317 Da), the class IIa bacteriocin hiracin-JM79 (7,737 Da), and an enterocin A (13,264 Da). Strain 24FS is α-hemolytic (11), which is consistent with the observed presence of *yqfA*, encoding hemolysin III. It also has high adherence and biofilm-formation capacity (12), correlating with the presence of virulence-related adherence/biofilm genes, namely, *gelE* (gelatinase), *esp* (gene encoding a surface protein), *ace* (collagen adhesin), *cvfB* (conserved virulence factor B), and *ebpABC* (pili-encoding genes). The disk diffusion method (13) showed that the strain is resistant to chloramphenicol, tetracycline, and erythromycin but sensitive to penicillin and streptomycin, which is consistent with the presence of corresponding resistance genes (*cat*, chloramphenicol; *tetA*, *tetC*, *tetL*, *tetR*, and *ykkCD*, tetracycline; and *ermB*, erythromycin), as revealed using the Comprehensive Antibiotic Resistance Database (CARD) (14). Genes conferring resistance to aminoglycosides [ANT(6)-la and APH(3′)-IIIa] and fluoroquinolones (*gyrA* and *gyrB*) were also detected, although the corresponding resistance was not tested. In addition, genes mediating iron transport were identified, including *feuABC* (ferric iron uptake), *hmuUV* (haem uptake), *feoAB* (ferrous iron uptake), and *fetAB* (iron export).

The CRISPR finder tool (15) identified two CRISPR elements with five or nine direct repeats of 36 bp, with one associated with *cas-*family proteins. A conjugative Tn*916* transposon, associated with tetracycline resistance *tet*(M), was also identified. In addition, a class II integron with three antibiotic-resistance gene cassettes (*dfrA1*, dihydroflorate reductase; *sat2*, streptothricin acetyltransferase; and *aad1*, aminoglycoside adenyltransferase) was found and predicted to confer resistance to trimethoprim, streptothricin, and streptomycin, respectively. Two plasmids (16) were identified, namely, pS194 and pAD1 (4.3 and 60 kb, respectively). The PHASTER tool (17) identified one intact (PHAGE_Entero_vB_IME197, 57.1 kb), one incomplete (PHAGE_Entero_vB_IME197, 14.6 kb), and two questionable (PHAGE_Entero_phiFL1A, 38.8 kb; and PHAGE_Brocho_NF5, 14.2 kb) prophage regions. One putative integrative conjugative element (ICE) (T4SS) of 431,826 bp was identified by ICEfinder (18) containing a type IV secretion gene cluster. Thirty insertion sequence (IS) elements were predicted with IS finder (19).

These genomic data assist understanding of the emergence of antibiotic resistance and virulence in nonclinical E. faecalis isolates.

### Data availability.

This whole-genome shotgun project has been deposited in DDBJ/ENA/GenBank under the accession number PGCH00000000. The version described in this paper is the first version, PGCH01000000. The raw sequencing data are available in the Sequence Read Archive (SRA) database under the accession number SRR8433214.
